# Compound heterozygous protein C deficiency with pulmonary embolism caused by a novel PROC gene mutation: Case report and literature review

**DOI:** 10.1097/MD.0000000000031221

**Published:** 2022-10-21

**Authors:** Zhaorui Zhang, Zhen Yang, Mei Chen, Yuzhu Li

**Affiliations:** a Department of Respiration, Eighth Medical Center of Chinese PLA General Hospital, Beijing City, People’s Republic of China; b Kingmed Diagnostic Group Co.Ltd, Guangzhou City, Guangdong Province, People’s Republic of China; c Department of Respiratory Disease, Hainan Hospital of Chinese PLA General Hospital, Hainan Province, People’s Republic of China.

**Keywords:** compound heterozygous mutation, protein C deficiency, pulmonary embolism

## Abstract

Protein C is an anticoagulation agent, and protein C deficiency results in vascular thrombosis disease. Hereditary protein C deficiency is a risk factor for pulmonary embolism in adults. Pathogenic variants of the Protein C, Inactivator Of Coagulation Factors Va And VIIIa (PROC) gene which encodes protein C have been identified as a cause of protein C deficiency.

**Patient concerns:**

We describe a patient with a novel mutation in the PROC gene who was diagnosed with pulmonary embolism in a Chinese family.

**Diagnosis:**

According to the results of the pulmonary computed tomography angiography (CTA) and the level of blood protein C, the patient was diagnosed with pulmonary embolism caused by protein C deficiency.

**Interventions:**

Whole-exome sequencing (WES) was performed for the molecular analysis.

**Outcome:**

The results of patient’s deoxyribonucleic acid revealed a heterozygous mutation (c.237 + 5G > A) in intron 3 of the PROC gene. His father also harbored the same mutation in the PROC gene. We also reviewed the protein C deficiencies caused by PROC gene mutations in cases.

**Lessons:**

A novel mutation in intron 3 of PROC gene has not been previously reported in patients with pulmonary embolism caused by protein C deficiency. After anticoagulation therapy, the patient recovered, and CT showed resolution of the thrombosis. Pulmonary embolism may be caused by protein C deficiency and the rare compound heterozygous mutation in intron 3 of the PROC gene could cause protein C deficiency via impairment of the secretory activity of protein C.

## 1. Introduction

Pulmonary embolism (PE) is responsible for more than 100,000 cardiovascular disease-related deaths annually in the United States and is the third leading cause of cardiovascular mortality.^[1]^ It has been estimated that only 7% of the patients with PE who died were correctly diagnosed before death.^[2]^ Genetic abnormalities in proteins involved in the coagulation pathway leading to hypercoagulability have been found to be the cause of thrombophilia disease. Factor V Leiden (FV Leiden) and prothrombin G20210A gene mutations are highly prevalent in the Caucasian population. In contrast, in the Asian population, protein C (PC) and protein S (PS) deficiencies have a higher prevalence.^[3]^ PC is a vitamin K-dependent plasma zymogen that is synthesized mainly in the liver and plays an important roles in anticoagulation and fibrinolysis by inactivating the blood coagulation factors Va and VIIIa.^[4]^ Protein C is encoded by the Protein C, Inactivator Of Coagulation Factors Va And VIIIa (PROC) gene on chromosomes 2q13–q14 and is composed of 9 exons and 8 introns.^[5]^ Several studies have reported the spectrum of PROC mutation, and at least 300 mutations have been reported to date.

Here, we reported a patient diagnosed with pulmonary thromboembolism associated with protein C deficiency caused by a novel PROC gene mutation that has not been previously reported before.

## 2. Method

### 2.1. Ethical approval and consent for publication

The PROC gene mutation analysis was performed in accordance with the ethical committee of the PLA General Hospital. Peripheral blood samples were also obtained from his parents after obtaining consent for deoxyribonucleic acid analysis.

### 2.2. Detection of mutation

We use whole-exome sequencing (WES) in panel to determine the possible mutations. If possible pathogenic mutations were found, his parents mutations wound be identified.

### 2.3. Review of literature

We searched PubMed for PROC gene mutations and protein C deficiency in English. Articles that lacked genetic analysis and basic patient information were excluded, and 26 studies were included in our study.^[6–31]^

## 3. Case report

A 23-year-old man was admitted to Hainan Hospital with headache for 24 days and hemoptysis for 4 days in 2018-3-12. The patient had headaches since February 2018 and hemoptysis since 2018-3-8. The patient was previously healthy and had a negative medical history. Magnetic resonance imaging of the brain was normal, and ultrasonography of both cephalic veins showed thrombosis.

Physical examination revealed a temperature of 37.1 °C, pulse of 88 beats/minute, respiratory rate of 20 breaths/minute, and blood pressure of 130/80 mm Hg. The patient’s state of consciousness was poor. Crackles were heard in both lungs. His heart rate was normal, and no murmur was heard in the auscultation area of each valve. The abdomen was soft with no tenderness or rebound pain, and the liver and spleen ribs were not observed. Muscle strength was normal, and meningeal stimulation was negative.

**Figure 1.**
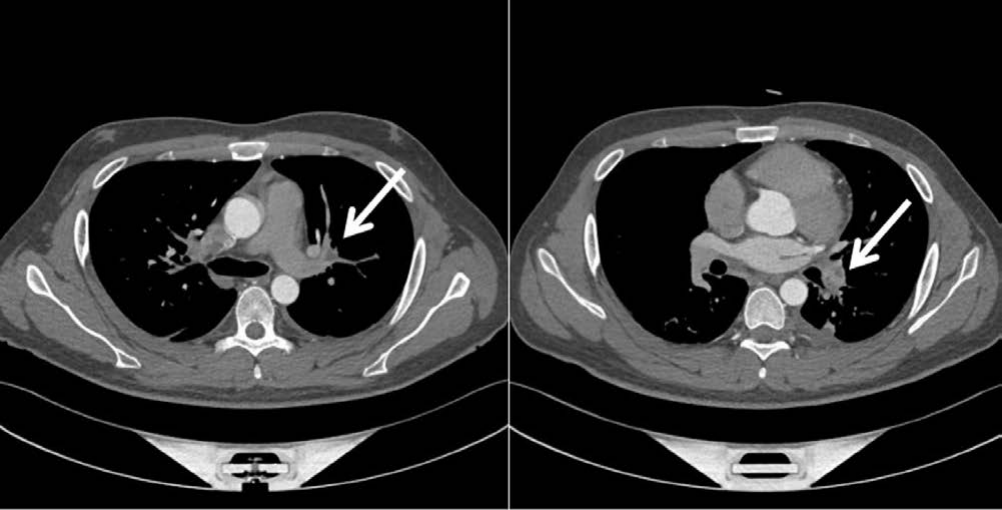
CTA of the pulmonary artery (the white arrow shows the embolism). CTA = computed tomography angiography.

Laboratory tests showed the following results: white blood cell (WBC), 9.48 × 109/L (normal range: 3.5–10109/L), neutrophils: 0.747 (normal range: 0.4–0.75), platelets: 507 × 109/L (normal range:100–300109/L), C-reactive protein 13.15 mg/dL (normal range:0–8 mg/L), interleukin-6 34.82 pg/mL (0.373–0.463 pg/mL), procalcitonin 0.040 ng/mL (normal range: <0.5 ng/mL), D-dimer: 3654 ng/mL (normal range <500 ng/mL), international normalized ratio (INR):1.46 (normal range: 0.8–1.5), prothrombin time, 15.7 s (11–13 s), fibrinogen level: 7.16 g/L (normal range: 2–4 g/L). We further performed computed tomography angiography (CTA) of the pulmonary artery, which revealed an embolism in the middle segment of the left pulmonary artery (Fig. 1).

For the patients, a young male adult with no history of illness, we further tested the anticoagulation factors, protein C, and protein; functional protein S (PS) activity of 112.4% (normal range: 60%–130%) and PC activity of 44.6% (normal range: normal range: 70%–140%) were measured using the StaClot (Diagnostic Stago Inc, Parsippany, NJ) PS and PC activity assay, which indicated a diagnosis of PC deficiency.

**Figure 2.**
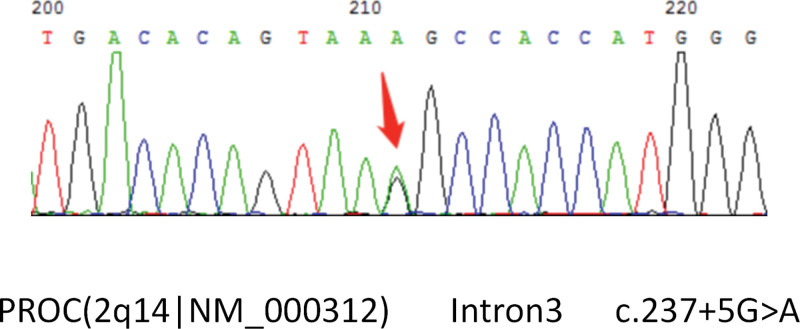
The patient with compound heterozygous mutations (c.237 + 5G > A).

**Figure 3.**
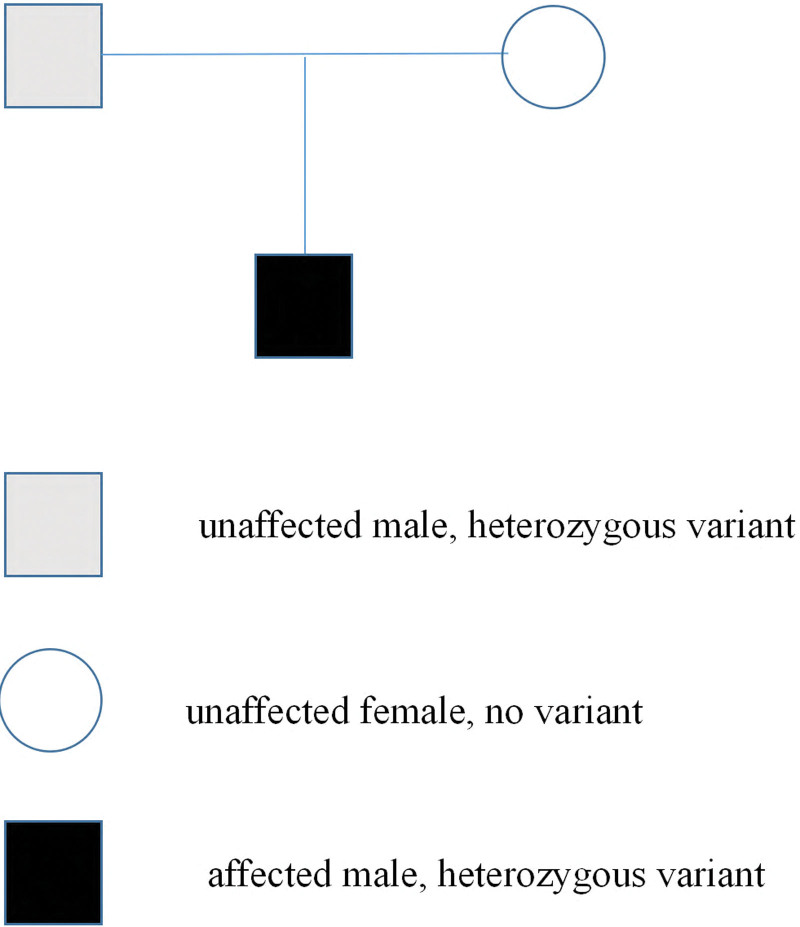
Family genetic analysis of the patient and his parents.

Molecular analysis was performed by targeting inherited diseases presenting as vasculitis or coagulopathy using next-generation sequencing. A heterozygous pathogenic variant located on intron 3 of the PROC gene (c.237 + 5G > A(2q14|NM-000312)) were identified (Fig. 2). His father was also a carrier of the c.237 + 5G > A mutation, while his mother was normal without a mutation in the PROC gene. Therefore, we deduced that the mutation was inherited from her father (Fig. 3).

**Figure 4.**
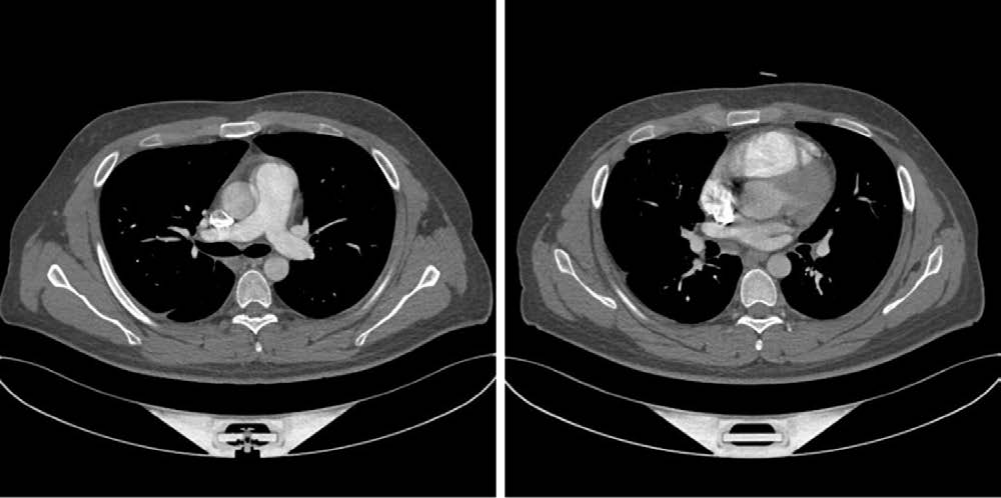
CTA of the pulmonary artery. CTA = computed tomography angiography.

The patient was treated with low-molecular-weight heparin (LMWH) anticoagulation at 14,000 IU per day (100 IU/kg) based on a weight of 70 kg. One month after treatment with LMWH, hemoptysis and chest pain were relieved. Subsequently, rivaroxaban (20 mg per day) was administered. The patient was discharged after taking rivaroxaban for 15 days without any adverse effects and continued taking rivaroxaban out of hospital. The symptoms of headache and hemoptysis disappeared, and reexamination of CTA of the pulmonary artery showed that the embolism had disappeared (Fig. 4). We followed up the patient monthly for 1 year, and no symptoms of thrombosis occurred.

## 4. Discussion and Conclusions

PC plays an important roles in anticoagulation and fibrinolysis and is often associated with pulmonary embolism. The incidence of heterozygous protein C deficiency is approximately 0.14% to 0.50% of the general population,^[32]^ and homozygote and compound heterozygote for PROC gene mutation are rare disorders (prevalence approximately 1 per 200,000–400,000 individuals). There have been only been a few case reports of pulmonary embolism caused by PROC gene mutation.^[14,33]^ In this case, we reported the case of an adult male with pulmonary embolism with a novel mutation in the PROC gene that had not been previously reported before. We further tested the genetic condition of the patients. His father had the same mutation; however, he did not show any evidence of deep venous thrombosis or pulmonary embolism. Therefore, we deduced that the intron 3 mutation (c.237 + 5G > A) in the PROC gene may be an autosomal recessive mutation, and patients with this mutation may or may not present with protein C deficiency.

More than 300 mutations that disrupt protein C levels have been identified. Our article reviewed 26 cases showing that protein C deficiency with a PROC mutation is rare (Table 1). These cases include 15 infants most of whom had homozygous mutation with poor prognosis. The symptoms of them often present as severe purpura fulminans.^[8,11,12,17,20–22,27,28,31]^ These PROC mutations are located in exons 4 to 9 and intron 8. Exons 7 and 9 were most frequent involved, most of the mutations were point mutations, 5 cases showed a del mutation of the PROC gene,^[8,9,15,17,21]^ one case showed a frameshift mutation of duplicated of c246_247.^[24]^ The mutation in 20 cases were mutation in exons, which can directly change the expression of proteins. For example, changes in c.1152C > G leads to p.N384K (replacement of asparagine by lysine) and c.1207G > T leads to p.G403W (glycine by tryptophan).^[10]^ Changes in the homozygous missense mutation, c.1198G > A leads to the replacement of Gly with Ser in the 400 amino acid.^[11]^ However, not all cases had exon mutations; 1 case reported that a patient suffered from deep venous thrombosis at the age of 26 years, and genetic analysis showed a transition at nucleotide 7054 in intron 7 (7054G to A). The 7054 mutation G to A caused the amplified exon 8 fragment to cause the R87H mutation^[19]^ and led to protein C deficiency and deep venous thrombosis. Interestingly, our case also had a mutation of introns in the PROC gene, resulting in protein C deficiency. This suggests that introns may not be nonsense gene sequences; they may affect the expression of upstream and downstream exons. However, the exact mechanism of intron 3 mutation (c.237 + 5G > A) of the PROC gene requires further study to reveal its function.

**Table 1:** Genetic features of patients with PROC.

Case	Age	Gender	Variant in PROC	Positions
1*	22	Male	c.237 + 5G > A	Intron 3
2^[6]^	38 yr	Male	c.580C > T	Exon 7
			c.970G > A	Exon 9
	26 yr	Female	c.820G > T	Exon 9
			c.889G > C	Exon 9
3^[7]^	27 yr	Male	G-to-C codon 297	Exon 9
	51 yr	Male	C-to-T codon 210	Exon 9
4^[8]^	7 mo	NM	T > C Promoter -1504	NM
	Birth	NM	c.340_346delinsATGCC	NM
	Birth	NM	c.829G > A	NM
5^[9]^	9 yr	Male	c.577_579delAAG	Exon 7
6^[10]^	45 yr	Male	Heterozygous	NM
			c.1152 C > G	
			c.1207G > T	
7^[11]^	8 mo	Male	c.1198G > A	
8^[12]^	10 d	NM	6246 G→A	Exon 7
	3 d	NM	Homozygous,3156, del C	Exon 5
9^[13]^	49 yr	Male	c.1015G > A	Exon 9
10^[14]^	29 yr	Male	Heterozygous T > C codon 106	
11^[15]^	40 yr	Male	c.577-579delAAG	Exon 7
12^[16]^	6 d	Male	Homozygous c.796 + 3A > T	Intron 8
13^[17]^	2 d	Female	Homozygous, deletion (GCGGGGCAGT between nucleo tides 7173–7182	Exon 8
14^[18]^	19 yr	Male	Heterozygous c.949C > T	Exon 9
15^[19]^	26 yr	Male	7054G→A	Intron 7
16^[20]^	13 yr	Female	335 GAC > TAC,	Exon 4
17^[21]^	28 wks	Male	c.574_576delAAG	Exon 7
18^[22]^	18 d	Female	Homozygous c.346G > T	NM
19^[23]^	3 d	Female	Heterozygous c.1015G > A	Exon 9
20^[24]^	28 yr	Male	c246_247dupCT	NM
21^[25]^	28 yr	Male	g.7271G > A	Exon 5
22^[26]^	63 yr	Male	C8516T	Exon 9
23^[27]^	10 d	Male	Homozygous T903C	NM
24^[28]^	6 d	Female	Homozygous c.1048A > T codon 350	NM
25^[29]^	20 yr	Male	Heterozygous 715_724delGGGGCAGTGC	Exon 8
26^[30]^	5 mo	Male	Homozygous (A-G)-12	NM
27^[31]^	4 d	Male	(C > T) codon 253	exon 8

NM = not mentioned in the article, PROC = Protein C, Inactivator Of Coagulation Factors Va And VIIIa.

* Case one is from this study.

In conclusion, we identified a novel heterozygous mutation in the PROC gene in a patient with protein C deficiency, which has not been previously reported. The patient was treated with LMWH and rivaroxaban for anticoagulation, with good progress. This article provide a more complete gene mapping with PROC gene in Chinese population. We hope that our report will further assist other clinicians in diagnosing and treating this rare disease.

## Acknowledgements

The authors gratefully acknowledge the patient and his family for allowing us to share their information.

## Author contributions

Zhaorui Zhang participated in the writing of the paper. Yuzhu Li participated in the study design of the work. Zhen Yang participated in the primary data acquisition and clinical analysis. Mei Chen participated in the genetic analysis of patients.

**Conceptualization:** Zhaorui Zhang, Zhen Yang, Yuzhu Li.

**Data curation:** Zhaorui Zhang, Zhen Yang, Mei Chen, Yuzhu Li.

**Formal analysis:** Zhen Yang, Yuzhu Li.

**Investigation:** Zhen Yang, Yuzhu Li.

**Methodology:** Zhen Yang, Yuzhu Li.

**Writing – original draft:** Zhaorui Zhang.

**Writing – review & editing:** Zhaorui Zhang.
